# Protecting Effects of Dexamethasone on Thymus of Rats with Severe Acute Pancreatitis

**DOI:** 10.1155/2007/72361

**Published:** 2007-12-17

**Authors:** Zhang Xiping, Chen Li, Lin Miao, Tian Hua

**Affiliations:** ^1^Department of General Surgery, Hangzhou First People's Hospital, Hangzhou 310006, Zhejiang Province, China; ^2^Department of General Surgery, Second Affiliated Hospital Medical College, Hangzhou 310003, Zhejiang Province, China; ^3^Zhejiang University of Traditional Chinese Medical, Hangzhou 310053, Zhejiang Province, China

## Abstract

*Purpose*. To study the protecting effects of dexamethasone on thymus of rats with severe acute pancreatitis (SAP). *Methods*. The SAP rats were randomly assigned to the model group and dexamethasone-treated group, the other normal healthy rats were assigned to the sham operation group. The rat survival, thymus pathological changes, apoptotic index, as well as expression levels of NF-κB, P-selectin, Bax, Bcl-2, and Caspase-3 protein of all groups were observed, respectively, at 3 hours, 6 hours, and 12 hours. The contents of amylase and endotoxin in plasma as well as the contents of TNF-α, ${\text{PLA}}_{2}$, and NO in serum were determined. *Results*. There was no marked difference between the model group and treated group in survival. The contents of different indexes in blood of treated group were lower than those of the model group to various degrees at different time points. The thymus pathological score was lower in treated group than in model group at 12 hours.The treated group in Caspase-3 protein expression of thymus significantly exceeded the model group at 12 hours. The apoptotic index was significantly higher in treated group than in model group. *Conclusion*. Dexamethasone has protecting effects on thymus of SAP rats.

## 1. INTRODUCTION

Acute pancreatitis (AP) especially severe acute pancreatitis (SAP) is a dangerous acute abdomen among diseases of digestive system. In its early stage, a big amount of inflammatory mediators will be released and activated to cause excessive immune response, resulting in cascade inflammatory injury or in severe cases systemic inflammatory response syndrome (SIRS) and multiple organ dysfunction syndrome (MODS) [1–3]. As a basic physiological mechanism of body to maintain normal morphous and function, apoptosis has been found in multiple organs in systemic SAP complications [4–6]. At present, the sound therapeutic effects of large dose of dexamethasone on SAP has been demonstrated but further study still should be conducted for its therapeutic mechanism. There are still few reports on thymus pathological changes during SAP all over the world [7]. In this experiment, the inflammatory mediators in blood, thymus apoptosis, and protein expression of NF-κB, P-selectin, Bax, Bcl-2, and Caspase-3 upon the onset of rat SAP have been studied to discuss the protecting effects of dexamethasone on SAP complicated thymus injury and its mechanism. The tissue microarray has also been applied to the pathohistological examination of pancreatitis to improve the study efficiency, which was first reported in this article around the world.

## 2. MATERIALS AND METHODS

### 2.1. Materials

Clean grade healthy male Sprague-Dawley (SD) rat in 250–300 g of body weight purchased from the Experimental Animal Center of Medical School, Zhejiang University (Hangzhou, China). Sodium taurocholate and sodium pentobarbital purchased from Sigma-Aldrich (Mo, USA). Dexamethasone injection purchased from Zhejiang Xinchang (Shaoxing, China). The full automatic biochemical analyzer was used to determine the plasma amylase level (U/L). Plasma endotoxin tachypleus amebocyte lysate kit was purchased from Shanghai Yihua Medical Science and Technology Corporation (Institute of Medical Analysis in Shanghai, China), the calculation unit for content is EU/mL. The serum nitrogen monoxidum (NO) kit was purchased from Nanjing Jiancheng Bioengineering Research Institute (Shengzhen, China), the calculation unit is μmol/L. The TNF-α ELISA kit was purchased from Jingmei Bioengineering Corporation (Hangzhou, China), the calculation unit for content is pg/mL (ng/L). The serum secretory phospholipase A_2_ enzyme Assay ELA kit (PLA_2_) was purchased from RdD system Ins and the calculation unit is U/mL. The NF-κB, Bax, Bcl-2, and P-Selectinantibody were purchased from Santa Cruz Company (Calif, USA). Caspase-3 antibody was purchased from NeoMarkers Company (Calif, USA), DNA nick in situ end-labeling (TUNEL) kit purchased from Takara Company. The above determinations were all operated according to the instructions of the kits.

### 2.2. Methods

#### 2.2.1. Animal grouping

90 clean grade healthy male SD rats were prepared into the SAP models via the improved Aho’s method [8] and randomly divided into the model group (45 rats) and dexamethasone-treated group (45 rats). Another 45 were selected to be the sham operation group. In the next step, the above groups were randomly divided into the 3hours, 6 hours, and 12 hours groups with 15 rats in each group. The dexamethasone-treated group was injected with dexamethasone injection via vena caudalis, 0.5 mg/100 g body weight and single administration 15 minutes after successful preparation of SAP model. The sham operation group after receiving abdomen opening performed pancreas and duodenum turning over and finally abdomen closing. The sham operation group and model group were injected with the saline of the same volume via vena caudalis 15 minutes after the operation [9, 10].

#### 2.2.2. Animal model preparation

Fasting but water restraining were imposed on all rat groups 12 hours prior to the operation. The rats were anesthetized by intraperitoneal injection of 2% sodium pentobarbital (0.25 mL/100 g) after which the rats are laid and fixed, and routine shaving, disinfection, and draping were performed. Model group: after entering abdomen via median epigastrium incision, confirmed the bile-pancreatic duct and hepatic hilus common hepatic duct, disclosed the pancreas, identified the duodenal papilla inside the duodenum duct wall, and then used a No. 5 needle to drill a hole in the mesenterium avascular area. After inserting a segmental eqidural catheter into the duodenum cavity via the hole, inserted into the bile-pancreatic duct toward the direction of papilla in a retrograde way, used the microvascular clamp to nip the catheter head temporarily and meanwhile used another microvascular clamp to temporarily occlude the common hepatic duct at the confluence of hepatic duct. After connecting the eqidural catheter end with the transfusion converter, transfused 3.5% sodium taurocholate 0.1 mL/100 g by retrograde transfusion via the microinjection pump at the speed of 0.2 mL/min. Stayed for 4 minutes after injection and removed the microvascular clamp and epidural catheter. After checking for bile leakage, sutured the hole in the duodenum lateral wall. Used the disinfected cotton ball to absorb up the anesthesia on the abdominal cavity and close the abdomen [8].

#### 2.2.3. NF-κB, P-selectin, Bax, Bcl-2, and Caspase-3 protein expression

Applied tissue microarrays to prepare thymus microarray sections; adopted streptavidin peroxidase (SP) method for immunohistochemical staining; observed the NF-κB, P-selectin, Bax, Bcl-2, and Caspase-3 protein expression of thymus under light microscope, respectively, and carried out the comprehensive assessment according to the positive cell percentage: positive cell count < 10% means (-); positive cell count 10–20% means (+); positive cell count 20–50% means (++); positive cell count >50% means (+++) [9].

#### 2.2.4. Apoptotic index

Applied the tissue microarrays to prepare the thymus microarray sections; Adopted DNA nick in situ end-labeling (TUNEL) technology for staining. Observed the thymus apoptotic cells and calculated apoptotic index, respectively. The TUNEL staining technique was applied to observe the changes of thymus apoptotic cells and the apoptotic indexes were calculated. Apoptotic index = apoptotic cell count/total cell count × 100% [9].

#### 2.2.5. Pathological score standard of thymus

There was no pathological score standard of thymus around world. Therefore, we have made a quantitative scoring standard according to the thymus pathological changes of different experimental groups, see Table 1.

### 2.3. Observation indexes

#### 2.3.1. Survival

Examined the rat mortality at 3 hours, 6 hours, and 12 hours after operation and calculated the survival, observed the gross changes of thymus.

#### 2.3.2. Pathological changes

After mercy killing, rats anesthetized by sodium pentobarbital in batches, collected the thymus and fixed them according to the related requirements, observed the pathological changes of thymus after HE staining, and performed thymus pathological score according to the self made standards see Table 1.

#### 2.3.3. Different indexes in blood

The contents of plasma amylase and endotoxin, serum NO, TNF-α, and PLA_2_ were determined via blood sampling from heart.

#### 2.3.4. Different proteins expression and apoptotic index

To observe NF-κB, P-selectin, Bax, Bcl-2, and Caspase-3 protein expression and apoptotic index of thymus.

### 2.4. Statistical methods

 The statistical analysis was conducted to the arranged experimental results by applying the SPSS11.5 software. The Kruskal-Wallis test or variance analysis (only applied to PLA_2_) was applied to the three group comparison. The Bonfferoni test was also applied to comparison. There are statistical significances when *P* < .05**.**

## 3. RESULTS

### 3.1. Survival

Model group: mortality, respectively, was 0% (0/15), 0% (0/15), and 13.33% (2/15) at 3 hours, 6 hours, and 12 hours while survival was 86.67% all along. The sham operation group and dexamethasone-treated group survived at all time points with 100% survival while there was no marked difference between the model group and dexamethasone-treated group (*P* > .05) [8–10].

### 3.2. Comparison of plasma amylase content

The model group and dexamethasone-treated group significantly exceeded the sham operation group at all time points (*P* < .001). No marked difference between the dexamethasone-treated groupand model group at 3 hours and 6 hours (*P* > .05). The dexamethasone-treated group was significantly less than the model group at 12 hours (*P* < .01), see Table 2.

### 3.3. Comparison of plasma endotoxin content

The model group and dexamethasone-treated group significantly exceeded the sham operation group at all time points (*P* < .001). No marked difference between the dexamethasone-treated group and model group at 3 hours (*P* > .05). The dexamethasone-treated group was significantly less than the model group at 6 hours and 12 hours (*P* < .01), see Table 2.

### 3.4. Comparison of serum NO content

The model group and dexamethasone-treated group significantly exceeded the sham operation group at all time points (*P* < .001). The dexamethasone-treated group was significantly less than the model group at 3 hours and 12 hours (*P* < .01), see Table 2.

### 3.5. Comparison of serum TNF-α content

The model group and dexamethasone-treated group significantly exceeded the sham operation group at all time points (*P* < .001). No marked difference between the dexamethasone-treated group and model group at 3 hours (*P* > .05). The dexamethasone-treated group was significantly less than the model group at 6 hours and 12 hours (*P* < .05), see Table 2.

### 3.6. Comparison of serum PLA_**2**_ content

The model group and dexamethasone-treated group significantly exceeded the sham operation group at all time points (*P* < .001). The dexamethasone-treated group was significantly less than the model group at all time points (*P* < .001), see Table 3.

### 3.7. Pathological changes of thymus under light microscope of all groups

#### 3.7.1. Sham operation group

In sham operation group, histological findings of thymus at 3 hours, 6 hours, and 12 hours are consistent, thymus structure is normal, boundary between cortex and medulla is clear, cortex/medulla thickness ratio is around 2 *~*1 : 1, lobule visible, envelope is intact, epithelial cell is with “starry sky” changes scattered in cortex, condensed deep purple-blue cell nucleus; many slightly stained epithelial reticular cells in star shape and with multiple protuberances, with big nucleus and rich kytoplasm in medulla; medulla structure is more loose than cortex, few epithelial cell nucleus slightly stained, and some epithelial cell “vacuole like”.

#### 3.7.2. Model group

In model group, thymus cortex gradually thinned at 3 hours, 6 hours, and 12 hours, “starry sky” like epithelial cells with fragmented nucleus, fewer lymphocytes, slightly stained epithelial cell nucleus in medulla, and more chromatin-losing cells “vacuole like” than in normal group.

#### 3.7.3. Dexamethasone-treated group

In treated group, cortex slightly thinned, more “starry sky” like cortex epithelial cells than in normal group, much less lymphocytes, and many “vacuole like” epithelial cells in medulla.

### 3.8. Comparison of thymus pathological scores of all groups

The scores were higher in model group and treated group than in sham operation groupat different time points (*P* < .001). The score was lower in treated group than in model group at 12 hours (*P* < .05), see Table 4.

### 3.9. Changes of different proteins expression

**Figure 1:**
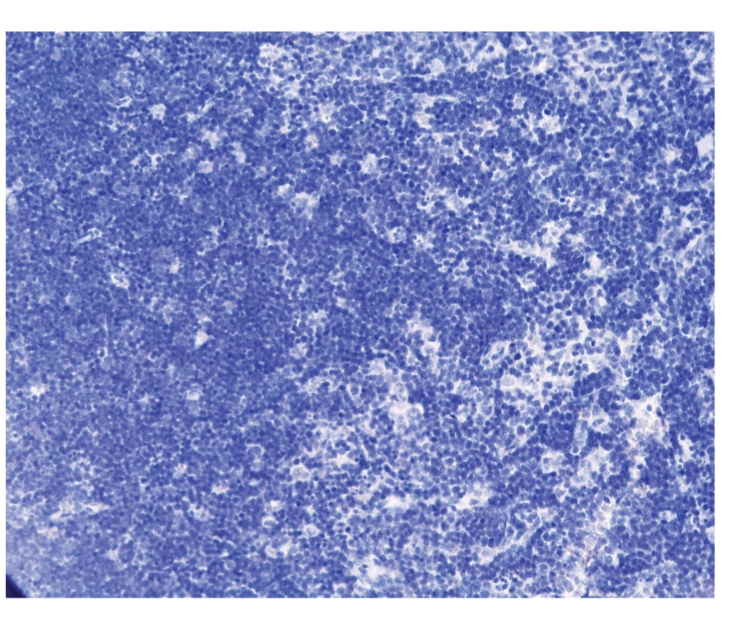
Treated group: 12 hours; NF-κB × 200.

The expression of NF-κB, P-selectin, Bax, and Bcl-2 in thymus was negative in all groups at different time points, see Figure 1.

### 3.10. Comparison of thymus apoptosis counts of all groups

**Figure 2:**
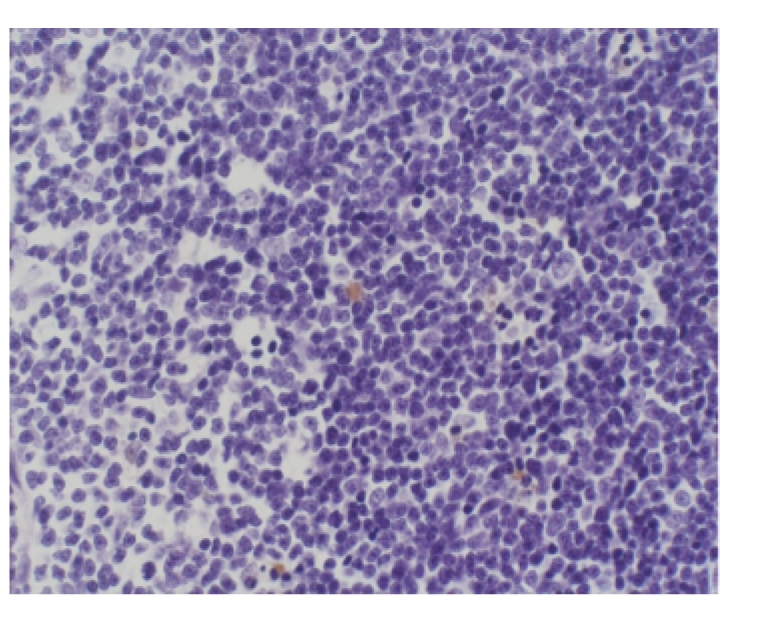
Sham operation group: 6 hours; Tunel × 200.

**Figure 3:**
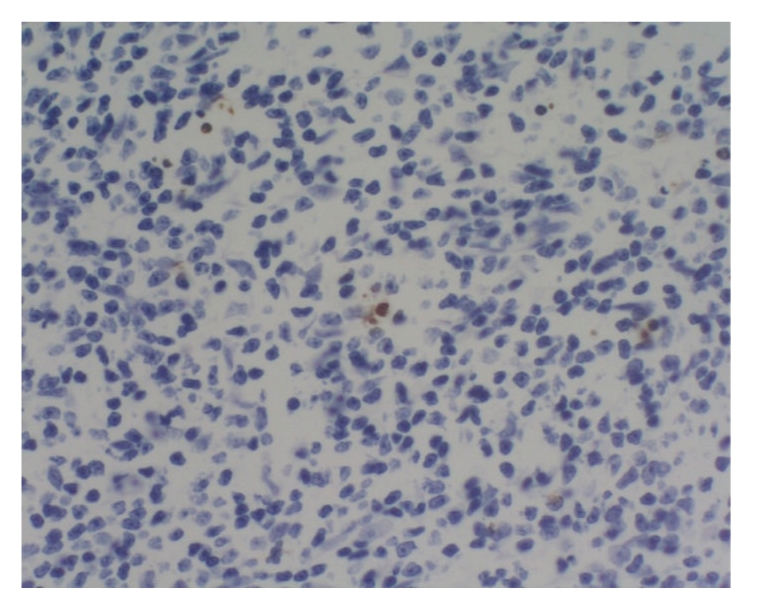
Sham operation group: 12 hours; Tunel × 200.

**Figure 4:**
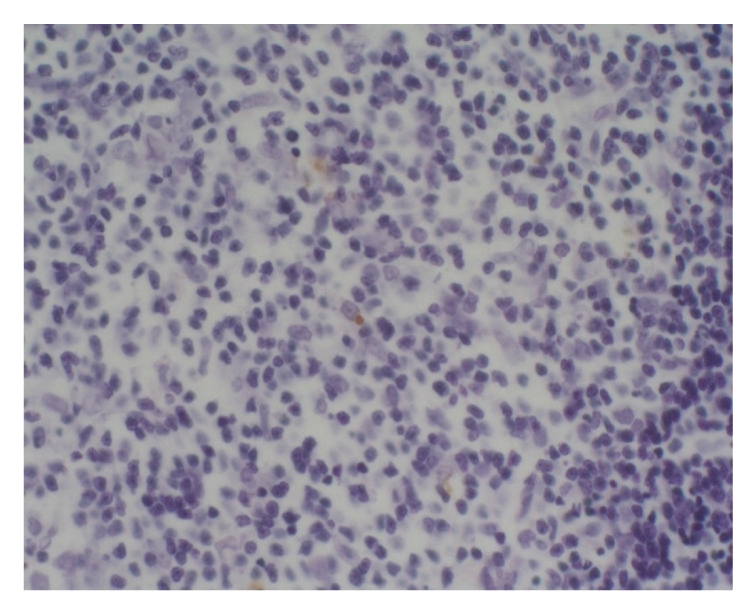
Model group: 12 hours; Tunel × 200.

**Figure 5:**
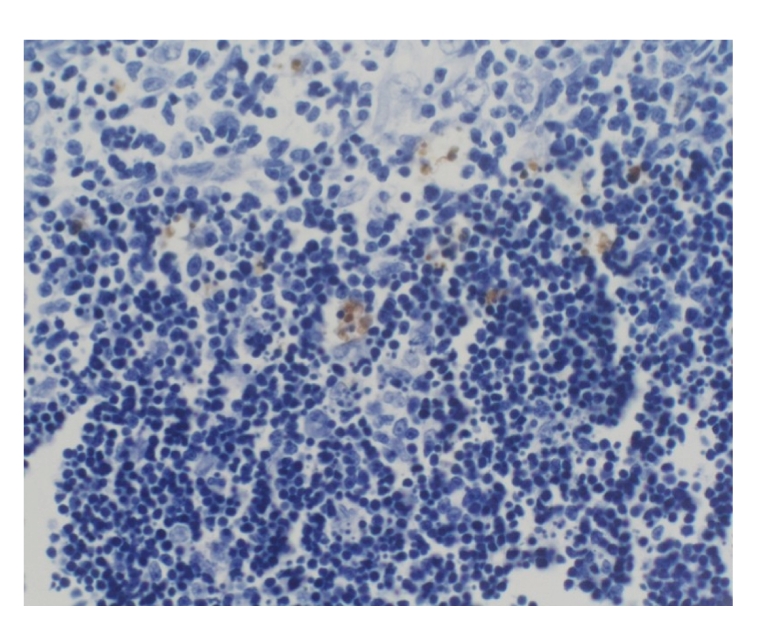
Treated group: 12 hours; Tunel × 200.

**Figure 6:**
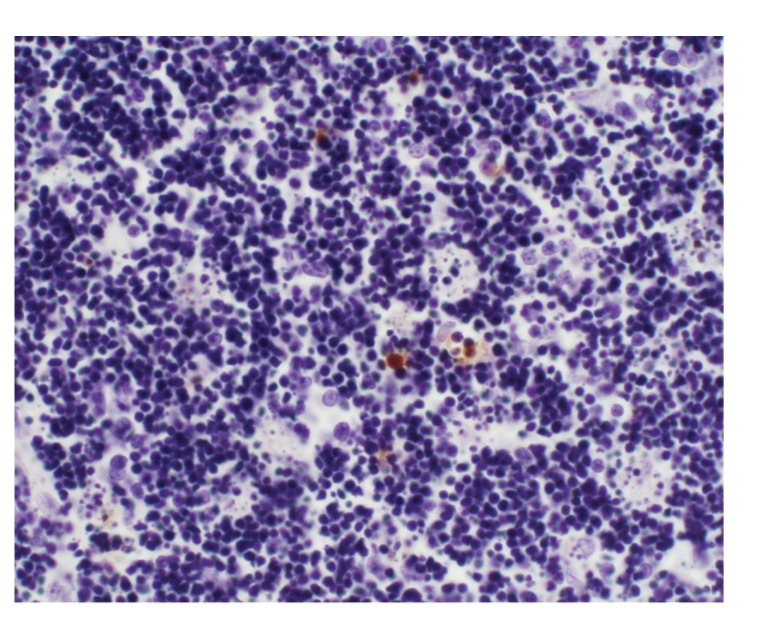
Treated group: 12 hours; Tunel × 200.

Lymphocytes were apoptotic cells of thymus. In sham operation group, apoptotic cells were found, respectively, in 2 and 1 rat at 6 hours and 12 hours, and the apoptotic index was between 10 per 10000 and 24 per 10000 as shown in Figures 2 and 3. In model group, apoptotic cells were found in 2 rats at 12 hours, and the apoptotic index was between 6 per 10000 and 8 per 10000 as shown in Figure 4. In treated group, apoptotic cells were found, respectively, in 6, 9, and 8 rats at 3 hours, 6 hours, and 12 hours, and the apoptotic index was between 2 per 10000 and 76 per 10000 as shown in Figures 5 and 6. The counts were higher in treated group than in sham operation group and model group at different time points (*P* < .05). There was no marked difference between model group and sham operation group (*P* > .05), see Table 5.

### 3.11. Comparision of Caspase-3 protein of thymus of all groups

**Figure 7:**
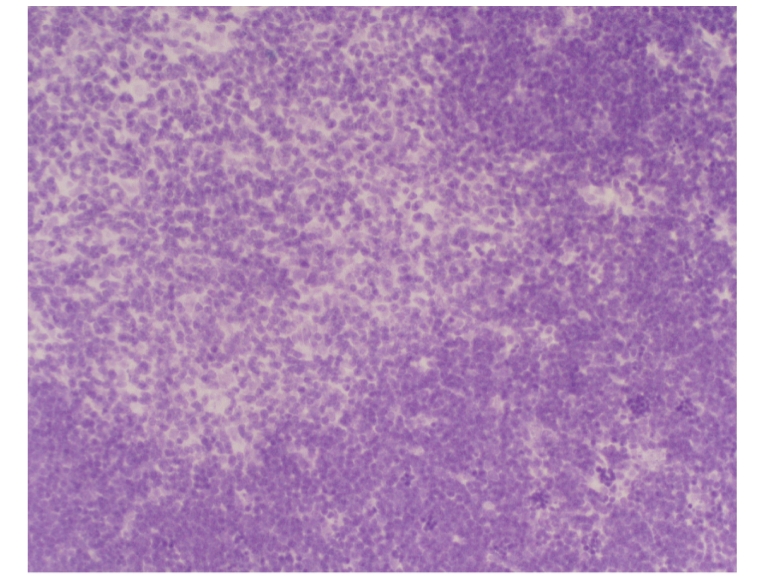
Sham operation group: 12 hours; Caspase-3 × 200.

**Figure 8:**
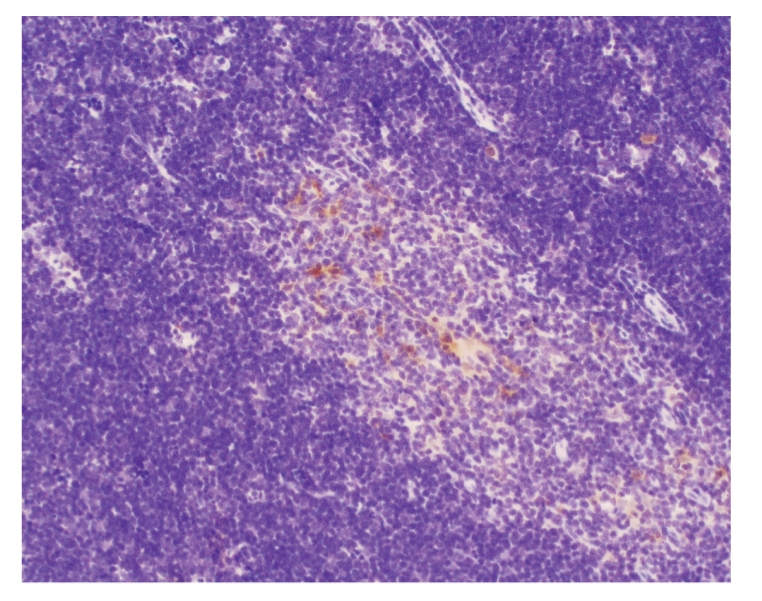
Model group: 12 hours; Caspase-3 × 200.

**Figure 9:**
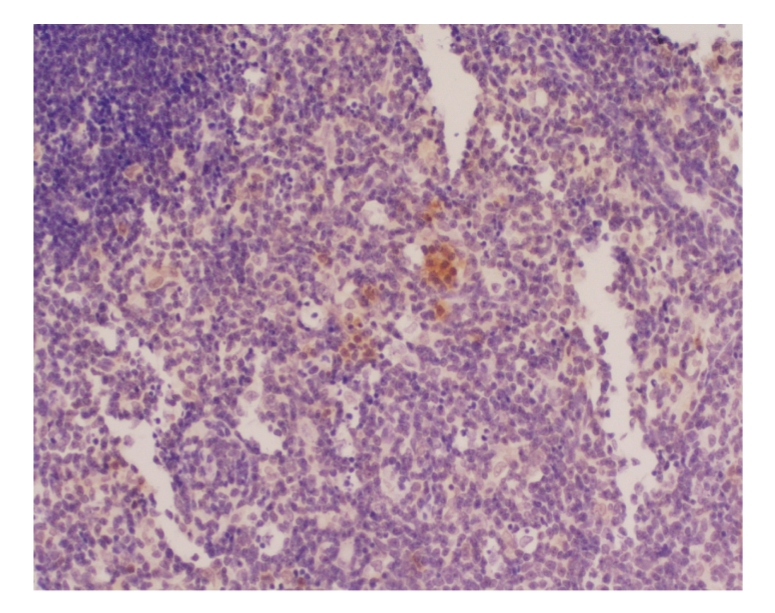
Treated group: 3 hours; Caspase-3 × 200.

**Figure 10:**
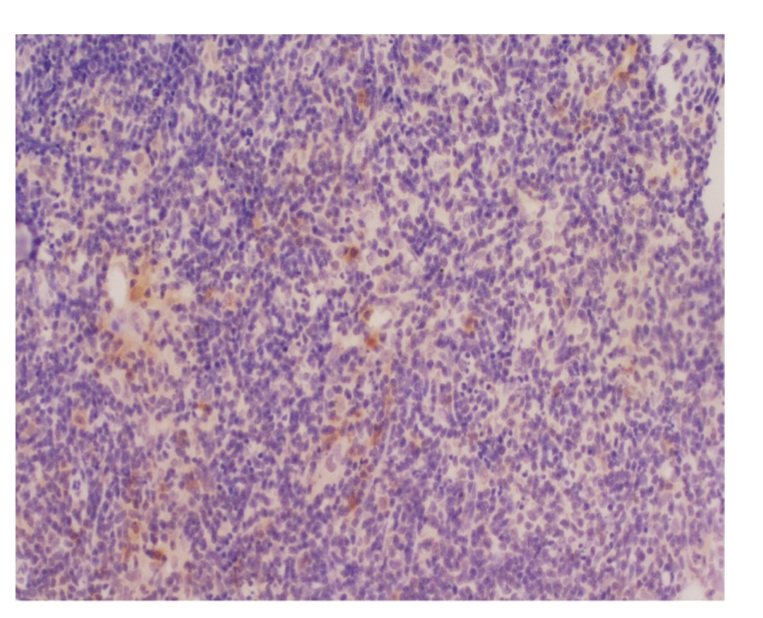
Treated group: 6 hours; Caspase-3 × 200.

The dexamethasone-treated group significantly exceeded the sham operation group at different time points (*P* < .01). The model group significantly exceeded the sham operation group at 3 hours and 6 hours (*P* < .05). The dexamethasone-treated group significantly exceeded the model group at 12 hours (*P* < .05), see Tables 6 and 7, and Figures 7–10.

### 3.12. Correlation analysis

The amylase of the model group was positively correlated with PLA_2_ at 3 hours (*P* < .05). The amylase of the model group was positively correlated with NO at 6 hours (*P* < .05). The NO of the dexamethasone-treated group was positively correlated with PLA_2_ (*P* < .05) and apoptotic indexes were negatively correlated with PLA_2_ at 3 hours (*P* < .05). The TNF-α content of the dexamethasone-treated group was positively correlated with PLA_2_ at 6 hours (*P* < .05). Apoptotic indexes of the dexamethasone-treated group were negatively correlated with PLA_2_ at 12 hours (*P* < .05).

## 4. DISCUSSIONS

When SAP occurs, pancreatins such as trypsin, pancrelipase, and amylopsin will be activated and excessively released [11], resulting in necrosis of pancreas and tissues around it. The absorbed necrotic tissue and bulk toxic substances will cause severe systemic inflammatory reaction. The inflammatory mediators are TNF-α, PLA_2_, NO, endotoxin, and so on. As one of the final common mediators in cascade reaction of inflammatory mediators [12], NO can be regarded as a study index for the changes of SAP state. TNF-α which is an important cytokine participates in SAP inflammatory cascade reaction [13, 14]. In SAP cases, endotoxin may reach tissues such as lung and liver due to intestinal mucosa damage [15]. The abnormal release and activation of PLA_2_ are closely related with SAP severity, and the PLA_2_ inhibitor can improve the pathological changes of SAP [16, 17]. The activation of NF-κB, which is a transcription factor participating in the regulation of inflammatory molecule expression and regulates inflammatory mediator expression, is the key initial step of inflammatory reaction [18, 19]. P-Selectin is a member of the family of cell adhesion molecule and is expressed in most architectonic blood vessels of the normal human body. However, the content is very low and the expression can be significantly increased when in acute inflammation [20, 21]. It is also an important indicator of inflammation [20, 22]. This study found that NF-κB and P-selectin were negative in all groups and showed that they were not related to the inflammatory reaction of thymus in SAP rats.

In this experiment, the plasma endotoxin content and contents of NO, TNF-α, and PLA_2_ in serum were all lower in treated group than in model group, and were negatively correlated with thymus pathological changes, demonstrating a close relation between SAP severity and content of inflammatory mediators.

Playing an important role in the onset, progression, and prognosis of AP, apoptosis can be categorized into gene-regulated and nongene-regulated apoptosis. There are direct and indirect gene regulations. Bax and Bcl-2 are two important components of the apoptosis regulation system, but in this experiment, Bax and Bcl-2 were negative and not irrelevant to apoptosis of thymus. Caspase-3 is one of the important proteases which can induce apoptosis and is also the final effect factor of the caspase cascade effect which is involved in apoptosis, and moreover it is at the core position in the process of cutting protease cascade. Caspase-3 is a marker of apoptosis and it is also the performer of apoptosis. It can destroy a variety of protease complex in cells with the digestive way and activate intranuclear nuclease to cause the DNA schizolysis form the DNA fragments, to undermine cell calcium pump function, to lead to the situation of intracellular calcium overload, and so on [23, 24]. Inhibiting Caspase-3 activity can reduce the occurrence possibility of apoptosis [25]. In this study, we found that the level of thymus Caspase-3 protein expression in the treated group was significantly greater than that of the model group at 12 hours. It is shown that the role of dexamethasone inducing thymus apoptosis may be relevant to expression of Caspase-3 protein in SAP.

In addition to be significant in inducing the indirect gene regulation of apoptosis, inflammatory mediators are important apoptosis participants during AP and their roles are nonneglectable. These cytoactive molecules are TNF-α, TGF, IL, NO, OFR, and so on [26–30]. In this experiment, the content of inflammatory mediators was lower in treated group than in model group, and the apoptotic index was higher in treated group than in model group indicating that apoptosis can be promoted by reducing the expression of inflammatory mediators.

Thymus as an important immunoregulation organ plays an important role in SIRS and MODS due to SAP. In this experiment, the expression of inflammatory mediators was lower in treated group than in model group, consistent with the pathological severity of thymus and negatively correlated with thymus apoptosis count, which demonstrated the protecting effects of apoptosis on thymus. Therefore, the author believes that just like those findings in SAP pancreas [31, 32] study, when necrosis and apoptosis coexist, the protecting effect can be achieved by inducing apoptosis if necrosis prevails. Both necrosis and apoptosis are death modes of injured cells [33]. What makes apoptosis substantially different from necrosis is that apoptosis will not release the harmful substance in lysosome or cause intense inflammatory reaction [34].

In AP/SAP, glucocorticoid (represented by dexamethasone) mainly inhibits the generation and/or effect of inflammatory mediators, enhances physical stress, improves microcirculation, alleviates endotoxemia, eliminates free radicals, and inhibits NO and NF-κB [35–37]. Dong et al. [38, 39] have found early that large dose of dexamethasone can more effectively treat SAP. In this experiment, large dose of dexamethasone has been administered. At present, the capacity of dexamethasone to induce thymus apoptosis has been confirmed [40–43]. According to the study on SIRS and MODS, thymus apoptosis or proliferation will cause immune function disorder [44]. We believe that the excessive immune response can be depressed by reducing immunocytes to inhibit the release of inflammatory mediators. Therefore, SAP complications can be alleviated by dexamethasone through induction of thymus apoptosis.

The method of sodium taurocholate injection through biliopancreatic duct to induce SAP rat model was first reported by Aho et al. [45]. It is a classic model preparation method extensively applied by the world researchers. We have found in practice [38] that ideal SAP rat models can be induced by 3.5% sodium taurocholate injected by minipump after retrograde puncturation of segmental eqidural cathete via duodenal papilla. This method features mild wound, convenient operation, and high achievement ratio.

It was found in this study that the rat survival was higher in treated group than in model group, the pathological changes were milder in treated group than in model group, and the apoptotic index was higher in treated group than in model group (*P* < .05), which demonstrates that dexamethasone can promote the apoptosis of thymus and protect it. It was found in the experiment that the thymus apoptosis count of treated group was positively correlated with PLA_2_ and the expression of amylase, NO, and TNF-α was also positively correlated with PLA_2_, which suggests that dexamethasone can alleviate thymus injury by lowering PLA_2_ content in SAP circulatory blood, inhibiting the secretion of amylase, and promoting the generation of cytokines such as TNF-α and NO as well as thymus apoptosis. The endotoxin was lower in treated group than model group, which demonstrates that dexamethasone might inhibit the excessive systemic inflammatory reaction and indirectly induce apoptosis to improve the thymus injury of SAP rats. It can be figured out that dexamethasone did not induce thymus apoptosis through regulating NF-κB, P-selectin, Bax, and Bcl-2 of thymus since their expressions were negative at different time points.

Tissue microarray, featuring high-throughput, multiple samples as well as being economical and time saving, error reduction, convenient for empirical control design, and capable for combining with other biological technologies [46], has been extensively applied to fields such as oncopathology and drug study [47–49]. The tissue microarray has been used for the pathological examination of pancreatitis study for the first time in this experiment. We have used a tissue microarray section machine to make holes 2.0 mm in diameter on receptor paraffin block, and we have combined supersensitive SP immunohistochemical method, TUNEL, and other techniques to determine the protein expressions of Bax, Bcl-2, and NF-κB genes of thymus cells as well as apoptosis counts. The satisfying results demonstrate that tissue sample of 2.0 mm diameter can lead to reliable and highly representative experimental results. It is also energy-, time-, and reagent-saving, convenient for control, and so on.

## Figures and Tables

**Table 1:** Pathological score standard of thymus.

Pathological score	Comparison of cortex and medulla thickness	Count of epithelial cell with necrosis in cortex	Count of epithelial cell with vacuole degeneration in medulla
1	Cortex is thicker or thinner than or as thick as medulla.	1–3	
1.5		1–3	1–3
2		4–10	
2.5		4–10	4–5
3		>10	
3.5		>10	>5
4		Flaky necrosis	
4.5		Flaky necrosis	>5

**Table 2:** Comparison of contents of different indexes in blood ($M\left(Q_{R}\right)$).

Groups index	Sham operation			Model			Dexamethasone-treated group		
	3 h	6 h	12 h	3 h	6 h	12 h	3 h	6 h	12 h
Amylase (U/L)	2038 (346)	2117 (324)	1725 (434)	7423 (2275)	8149 (1540)	9195 (1298)	6739 (2310)	7839 (2258)	7791 (1863)
Endotoxin (EU/mL)	0.015 (0.007)	0.015 (0.007)	0.016 (0.005)	0.035 (0.017)	0.055 (0.025)	0.059 (0.020)	0.030 (0.014)	0.040 (0.012)	0.042 (0.018)
NO (μmol/L)	10.0 (12.5)	15.0 (7.5)	15.0 (10.0)	72.5 (17.5)	62.5 (27.5)	70.0 (13.75)	50.0 (20)	60.0 (12.5)	45.0 (17.5)
TNF-α (ng/L)	3.30 (3.60)	4.90 (2.60)	3.70 (2.30)	46.13 (37.95)	77.54 (42.16)	67.30 (32.13)	38.40 (26.60)	58.30 (26.40)	38.70 (28.5)

**Table 3:** Comparison of PLA_2_ in serum ($\overset{¯}{X}\pm S$).

Groups	3 h	6 h	12 h
Sham operation group	18.70±4.40	16.70±3.83	18.52±11.31
Model group	103.69±20.82	119.85±17.74	121.29±17.01
Dexamethasone-treated group	53.96±15.40	67.75±27.95	65.27±26.21

**Table 4:** Comparison of pathological score of the thymus ($M\left(Q_{R}\right)$).

Groups	3 h	6 h	12 h
Sham operation group	0.0 (1.0)	0.0 (2.0)	0.0 (1.0)
Model group	2.0 (0.0)	2.5 (2.0)	3.0 (1.5)
Dexamethasone-treated group	2.0 (1.5)	2.5 (1.0)	2.5 (0.5)

**Table 5:** Comparison of apoptosis index of the thymus ($M\left(Q_{R}\right)$).

Group	3 h	6 h	12 h
Sham operation group	0.00 (0.00)	0.00 (0.00)	0.00 (0.00)
Model group	0.00 (0.00)	0.00 (0.00)	0.00 (0.00)
Dexamethasone-treated group	0.00 (0.06)	0.06 (0.12)	0.02 (0.30)

**Table 6:** Expression of Caspase-3 protein of the thymus.

Group	Time (h)	Cases	Pathologic grade			
			-	+	++	+++
Sham operation group	3	15				
	6	15	15			
	12	15	15			

Model group	3	15	10	5		
	6	15	10	5		
	12	13	10	3		

Treated group	3	15	8	7		
	6	15	6	9		
	12	15	5	10		

**Table 7:** Comparision of Caspase-3 protein of the thymus ($M\left(Q_{R}\right)$).

Group	3h	6h	12h
Sham operation group	0.00 (0.00)	0.00 (0.00)	0.00 (0.00)
Model group	0.00 (1.00)	0.00 (1.00)	0.00 (0.00)
Treated group	0.00 (1.00)	1.00 (1.00)	1.00 (1.00)
